# Third Harmonic Generation in Transparent Longitudinal Epsilon‐Near‐Zero Multilayers

**DOI:** 10.1002/nap2.70216

**Published:** 2026-07-22

**Authors:** Wallace Jaffray, Sven Stengel, Domenico de Ceglia, Neset Akozbek, Filippo Capolino, Marcello Ferrera, Michael Scalora, Maria Antonietta Vincenti

**Affiliations:** ^1^ Institute of Photonics and Quantum Sciences Heriot‐Watt University Edinburgh UK; ^2^ Department of Information Engineering Università degli Studi di Brescia Brescia Italy; ^3^ U.S. Army Space and Missile Defense Command Redstone Arsenal Alabama USA; ^4^ Department of Electrical Engineering and Computer Science University of California Irvine California USA

**Keywords:** epsilon‐near‐zero, harmonic generation, low‐index materials, nonlinear optics, phase‐locking

## Abstract

Epsilon‐near‐zero (ENZ) materials can dramatically enhance local optical fields, enabling nonlinear interactions at relatively low intensities. Yet, near their plasma frequency, isotropic ENZ media remain highly absorptive, limiting nonlinear operations that require good transparency. Longitudinal epsilon‐near‐zero (LENZ) metamaterials, characterized by a vanishing permittivity along the optical axis, provide a promising route to field enhancement while mitigating absorption losses and impedance mismatch. We experimentally show that an αSi/ITO multilayer with a LENZ resonance in the near‐infrared enables broadband high pump transmission while still supporting ENZ‐enhanced harmonic generation. We observe a strong third‐harmonic signal under conditions of significantly higher pump transparency than comparable ITO films demonstrating how nonlinear processes can be driven in a transparent multilayer geometry, avoiding the high‐loss pump conditions typically associated with isotropic ENZ operation. Measured third‐harmonic efficiency is comparable to values reported for isotropic ENZ films, whereas the multilayer architecture provides a potential route to reduce absorption‐induced heating at the pump wavelength. These results establish transparent LENZ (TLENZ) multilayers as a versatile platform for transparent, field‐enhanced nonlinear interactions, combining strong light–matter interaction with reduced pump losses.

## Graphical abstract

Transparent longitudinal epsilon‐near‐zero stacks enable field‐enhanced nonlinear light generation while preserving high near‐infrared pump transmission. Broadband third‐harmonic generation is experimentally observed demonstrating that TLENZ architectures can mitigate pump‐loss limitations and offer a promising platform for transparent nonlinear nanophotonics.

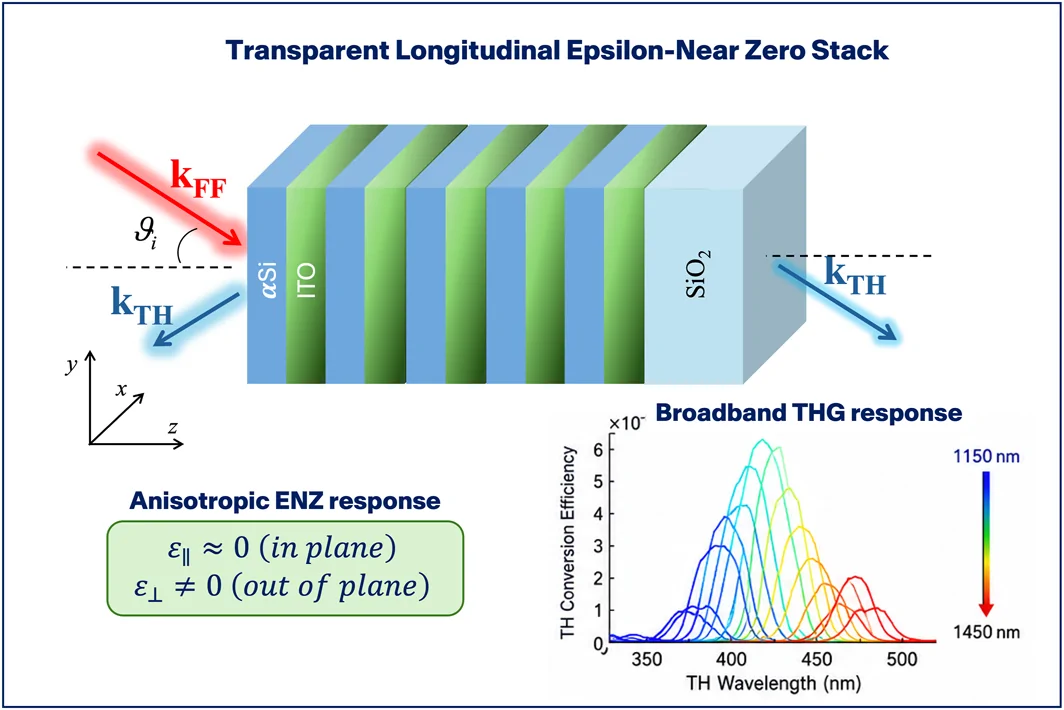

## 1 Introduction

Over the past decades, materials with vanishing permittivity, or epsilon‐near‐zero (ENZ) media, have captured interest across the photonics community for their ability to dramatically reshape light propagation. As the real part of the dielectric permittivity approaches zero, optical wavelengths effectively stretch toward infinity inside the medium, producing a nearly uniform internal phase, dramatically enhanced light–matter coupling, and exotic wave phenomena such as supercoupling and highly directive emission [1, 2, 3, 4, 5, 6, 7]. Beyond linear control of electro‐magnetic waves, both natural and engineered ENZ materials have emerged as powerful platforms to boost nonlinear optical interactions. In these systems, exceptional field confinement drives efficient harmonic generation and enables extreme nonlinear dynamics such as soliton formation, optical bistability, and ultrafast switching [8, 9, 10, 11, 12, 13, 14, 15, 16, 17, 18, 19, 20, 21, 22, 23, 24, 25, 26]. However, applications in isotropic ENZ media, such as transparent conducting oxides (TCOs), are limited by their relevant absorption near the plasma frequency [27, 28]. This is further aggravated as the conditions that produces maximum field enhancement coincides with a peak in the material absorption, thus preventing transparent nonlinear regimes. This limitation has driven interest in anisotropic ENZ materials, where only a single element of the permittivity tensor vanishes.

By alternating an ENZ material with another isotropic dielectric, one realizes a longitudinal ENZ (LENZ) medium in which the longitudinal component of the effective permittivity goes to zero whereas the transverse component remains finite [29]. LENZ designs are predicted to deliver much larger intensity enhancement and dramatically broader angular acceptance than their isotropic counterparts. Those advantages have been exploited to boost second‐harmonic generation efficiencies by more than an order of magnitude [30]. More broadly, extreme anisotropy promotes enhanced nonlinear interactions, especially in hybrid metal–TCOs architectures [31].

A similar strategy appears in metal–dielectric multilayers, where alternating thin metallic and dielectric films exposes the metal's intrinsic nonlinearity and avoids the heavy absorption of a bulk metal [32, 33, 34]. In these systems, careful engineering of the layer thicknesses allows nonlinear processes, such as harmonic generation, to be activated while maintaining significant optical transmission [35, 36, 37, 38]. This analogy underlines how judicious layering offers a general pathway to combine strong nonlinearity with transparency.

Despite these theoretical promises, experimental confirmation of LENZ‐enhanced nonlinear optics has remained elusive. Harmonic generation has been reported in anisotropic ENZ systems, such as phonon‐polariton AlN [39], but those studies did not directly probe harmonic generation in the LENZ regime or examine the interplay between nonlinearity and transparency.

In the present work, we close this gap by demonstrating THG in a transparent LENZ (TLENZ) multilayer. We design and fabricate an indium tin oxide (ITO) and amorphous silicon (αSi) multilayer stack that supports a longitudinal ENZ resonance in the near infrared while simultaneously showing broadband transparency. Thanks to the unique combination of TLENZ properties we can produce a strong nonlinear response without entering the high‐loss pump regime that limits the use of isotropic ENZ media. The observed third harmonic signal is in excellent agreement with our theoretical predictions both qualitatively and quantitatively. Moreover, efficient THG persists despite substantial absorption at the harmonic frequency, owing to phase‐locking between the pump and the third harmonic [40, 41, 42]. These results experimentally validate the ENZ route to strong nonlinear optics under transparent operation and establish TLENZ multilayers as a robust platform that combines intense light–matter interactions with low‐loss operation.

## 2 Effective Permittivity Response Analysis

We designed a ten‐periods stack composed of alternating layers of αSi and ITO (Figure 1a) to demonstrate the ability to combine a transparent linear response with efficient nonlinear processes. Individually, αSi and ITO are isotropic materials (see Supporting Information S1 for material ellipsometric data), when layered periodically with subwavelength thicknesses, the composite behaves as an effectively anisotropic system. Using Maxwell–Garnett effective medium theory, the transverse (in‐plane, x and y) and longitudinal (out‐of‐plane, z) components of the permittivity tensor are set by the constituent permittivities and the ITO volume filling ratio (FR), that is, the ratio between the ITO layer thickness and the periodicity of the stack. The transverse component is the arithmetic average of the permittivities, $\left[\varepsilon_{t}=FR\cdot \varepsilon_{\text{ITO}}+\left(1-FR\right)\cdot \varepsilon_{\alpha \text{Si}}\right]$, whereas the longitudinal component follows a harmonic‐type average $\left[\varepsilon_{l}=\frac{\varepsilon_{\text{ITO}}\cdot \varepsilon_{\alpha \text{Si}}}{FR\cdot \varepsilon_{\alpha \text{Si}}+\left(1-FR\right)\cdot \varepsilon_{\text{ITO}}}\right]$.

**FIGURE 1:**
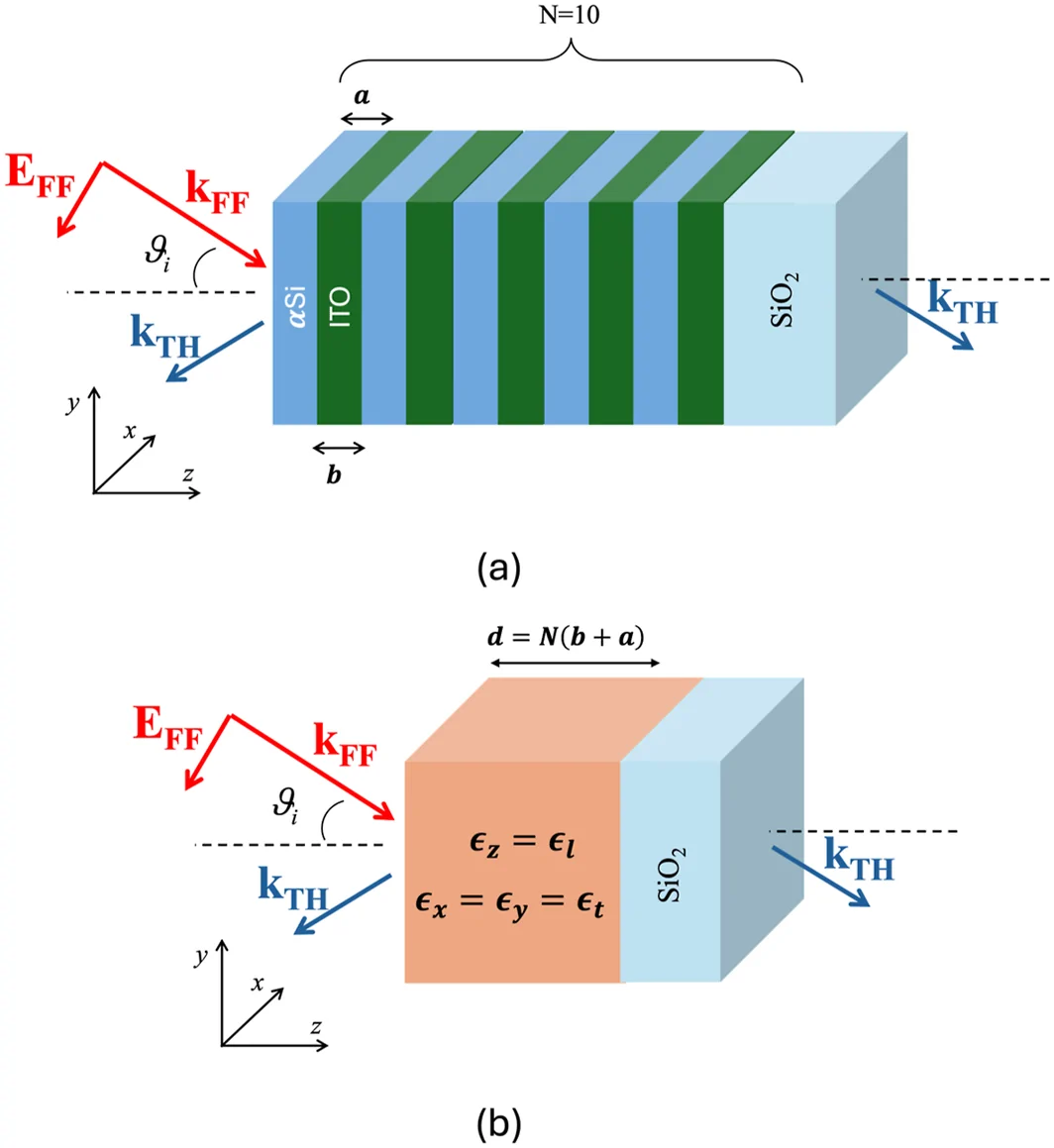
(a) Sketch of the multilayer and TM‐polarized pump excitation scheme. The multilayer is composed by *N* = 10 periods of αSi (thickness *a*) and ITO (thickness *b*) so that each period is equal to *p* = 50 nm for a total thickness of the multilayer *d* = *N* (*b* + *a*) = 500 nm. (b) Effective medium representation of the multilayer in panel (a).

In the near infrared, αSi is a high‐index dielectric with positive permittivity, whereas ITO crosses from positive to negative‐permittivity near its plasma frequency at 1248 nm. Consequently, small changes in the relative thicknesses within the multilayer can drive the effective longitudinal permittivity through zero, reversing the sign of the optical response and producing a large contrast between longitudinal and transverse components. Figure 2 shows the real and imaginary parts of the effective permittivities as a function of both wavelength and FR for a αSi/ITO stack with period *p* = 50 nm. The white dots in Figure 2a mark the wavelength‐FR combination that gives $\varepsilon_{l}=0$. Increasing the ITO filling ratio above 20% barely shifts the zero‐crossing wavelength, but the degree of anisotropy grows as the filling ratio decreases. Around the zero‐crossing wavelength (∼1260nm) the real part of the transverse effective permittivity $\varepsilon_{t}$ increases as the ITO fraction falls, while the imaginary parts $Im\left(\varepsilon_{t}\right)$ stays low and spectrally featureless, whereas $Im\left(\varepsilon_{l}\right)$ spikes just below the ITO plasma frequency for low FR (see Figure 2b). Overall, the control of $\epsilon_{t}$ with the FR around 1260nm seen Figure 2c offers an extra degree of freedom to control transparency and impedance matching, regardless of the materials above and below the multilayer.

**FIGURE 2:**
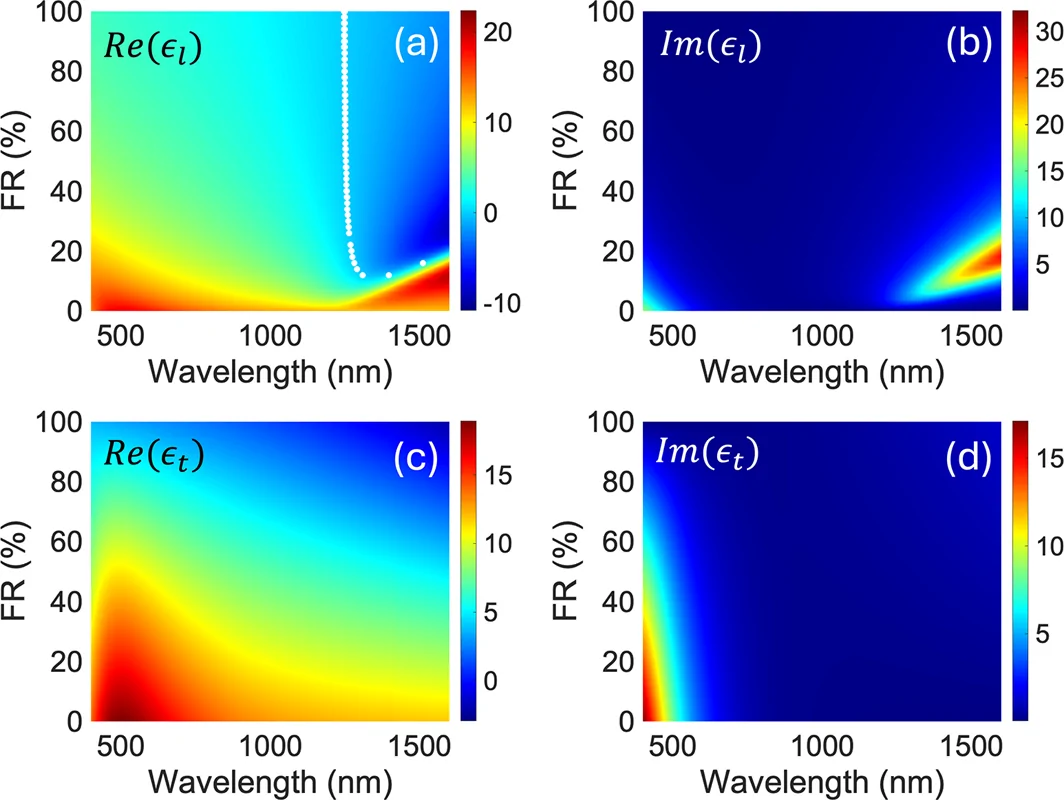
Effective permittivities of a αSi/ITO multilayer with period *p* = 50 nm as a function of wavelength and ITO filling ratio (FR). (a) Real part of the longitudinal permittivity $Re\left[\varepsilon_{l}\right]$, the white dots mark the wavelength/FR pairs where $Re\left[\varepsilon_{l}\right]=0$. The zero‐crossing wavelength remains largely unchanged above a 20% ITO FR, whereas decreasing the FR enhances the anisotropy. (b) Imaginary part of the longitudinal permittivity $Im\left[\varepsilon_{l}\right]$; a pronounced peak emerges just below the ITO plasma frequency at low FRs. (c) Real part of the transverse permittivity $Re\left[\varepsilon_{t}\right]$, which increases near the zero‐crossing wavelength (∼1260 nm) as the ITO FR decreases. (d) Imaginary part of the transverse permittivity $Im\left[\varepsilon_{t}\right]$, which remains low and spectrally featureless across the parameter range.

## 3 Combining Transparency With Efficient Nonlinear Response

Strong anisotropy, combined with a near‐zero longitudinal permittivity, can markedly boost the field intensity enhancement and broaden the angular acceptance compared to isotropic ENZ media as shown for LENZ [29]. Although such field enhancement is essential for driving nonlinear processes, our goal is also to retain high transparency at the pump wavelength, a condition unattainable in isotropic ENZ [27] or for a generic combination of materials that form a LENZ condition [31]. To meet both requirements, we fabricated a TLENZ made of 10 periods of αSi and ITO with a FR = 50% (for fabrication details see Supporting Information S1). Maxwell–Garnett theory gives effective permittivities at λ=1263nm of $\varepsilon_{l}=0.009+i2.396$ and $\varepsilon_{t}=8.992+i0.204.$ This results in a $|\varepsilon_{l}|\;\approx \;2.4\;$ and an anisotropy ratio ${\left|\epsilon_{t}\right|}^{2}/{\left|\epsilon_{l}\right|}^{2}\;\approx \;14$. According to Ref [29], anisotropy is responsible for field intensity enhancement (FIE). Therefore, under the condition $\left|\varepsilon_{l}\right|\ll \sin\;\theta_{i}$, where $\theta_{i}$ is the angle of incidence, one has $\text{FIE}\;\approx \;4{\left|\epsilon_{t}\right|}^{2}/{\left|\epsilon_{l}\right|}^{2}\cos^{2}\theta_{i}$; despite such ideal condition does not reflect our operating conditions, this formula readily shows in a simple way the beneficial effect of anisotropy. In other words, it is not important to have a small $\left|\epsilon_{l}\right|$ but rather a large ${\left|\epsilon_{t}\right|}^{2}/{\left|\epsilon_{l}\right|}^{2}$ as we have in the designed TLENZ. We then measured the stack's linear transmittance by utilizing a stable and calibrated white‐light source placed at almost normal incidence to the multilayer device to collect the normalized power transmission using a visible and near‐infrared spectrometer (Figure 3, red markers). We optimized multilayer transmission with a transfer‐matrix model, starting from 25 nm nominal thickness per layer (FR = 50%) and assuming the ITO and αSi dielectric permittivities identical to those retrieved with ellipsometric measurements on the single layers. The model was iteratively fitted to the measured transmittance by allowing each layer thickness to vary within ± 5 nm. Searching this restricted parameter space, we adjusted individual layer thicknesses to minimize the difference between simulated and experimental transmission, yielding an optimized stack that best reproduces the observed optical response (Figure 3, blue line). We stress that the measured transmittance exceeds 50% over a broad spectral range, despite the overall thickness of the stack and the material losses involved. The spectral peaks observed in the transmission originate from the multilayer geometry and can be understood as coupled‐cavity transmission resonances of the αSi/ITO stack. This mechanism is analogous to that occurring in metal–dielectric multilayers [31, 33, 34, 35, 38], where a sequence of coupled resonant cavities enables optical tunneling through the structure in spectral regions where the individual materials, namely ITO, would otherwise display significantly lower transmission (transmission of ITO films of different thicknesses can be found in Supporting Information S1). In other words, when the pump wavelength lies inside the transmission band, the field is not confined solely to the ITO layers; instead, it oscillates between ITO and αSi, creating regions where the local electric field intensity is comparable to or even exceeds that in ITO. In this regime, αSi becomes an active participant whose thickness determines not only the position and width of the broadband transmission region but also strongly impacts the nonlinear response of the stack (see also Section 4).

**FIGURE 3:**
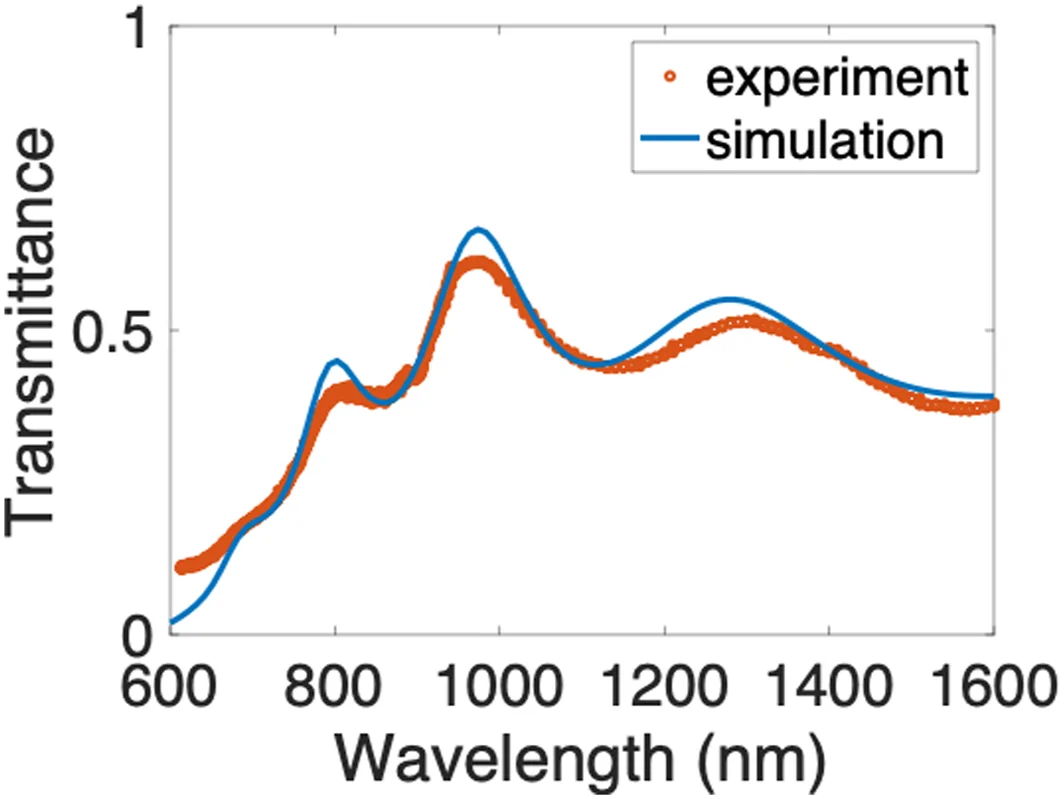
Transmittance of the 10 period αSi/ITO multilayer stack as measured (red markers) and simulated (blue line). The experimental curve has been fitted with an optimized stack with the following thicknesses: αSi 30 nm/ITO 20 nm/αSi 30 nm/ITO 30 nm/αSi 28 nm/ITO 30 nm/αSi 23 nm/ITO 30 nm/αSi 22 nm/ITO 30 nm/αSi 30 nm/ITO 25 nm/αSi 30 nm/ITO 30 nm/αSi 30 nm/ITO 30 nm/αSi 25 nm/ITO 30 nm/αSi 21 nm/ITO 20 nm.

We then moved forward to measure the TH signal by pumping the multilayer at 1250 nm. We first measured the angular response with a pump intensity I = 100 GW/cm^2^ and then we measured THG for increasing pump intensities. Specifically, the samples were excited using near‐infrared femtosecond pulses generated by an optical parametric amplifier (OPA) with wavelength tuneable in the 1150–1450 nm range. Pump power was adjusted to produce on‐sample intensities in the range of 15–150 GW/cm^2^. The beam was focused to a 0.35 mm diameter spot and operated at a 10 Hz repetition rate with 85 fs pulse duration. The generated TH signal was collected in transmission and analyzed spectrally using a calibrated detection system (see Supporting Information S1).

We solved the THG problem for a fixed TM‐polarized pump wavelength of 1250 nm using the finite element method (COMSOL Multiphysics) by implementing the nonlinear polarization terms for the pump at *ω* and its TH at 3*ω*. We assume an isotropic third‐order nonlinear response for both ITO and αSi, using the nonlinear susceptibilities and dispersion reported in ref. [43]. For the crystal symmetry considered, only three independent tensor components of $\chi^{\left(3\right)}$ contribute, so the nonlinear polarization reduces to its scalar form [44]. The nonlinear polarization induced by the pump field $E_{\omega }$ at *ω* and 3ω are written as follows:

(1) $$P_{\omega ,i}^{NL}=\epsilon_{0}\chi^{\left(3\right)}\;E_{\omega ,j}\;\left(3{\left|E_{\omega ,j}\right|}^{2}+2\;{\left|E_{\omega ,k}\right|}^{2}+2\;{\left|E_{\omega ,l}\right|}^{2}\right)$$

(2) $$P_{3\omega ,i}^{NL}=\epsilon_{0}\chi^{\left(3\right)}\;E_{\omega ,j}\;\left(\;E_{\omega ,j}^{2}+E_{\omega ,k}^{2}+E_{\omega ,l}^{2}\;\right)$$

These expressions include only pump–pump and pump–pump–pump mixing terms that drive self‐phase modulation of the fundamental frequency and for THG. Other frequency‐mixing contributions are neglected because they do not contribute significantly to the third order process. The model reproduces the measured third harmonic conversion efficiency both qualitatively and quantitatively when considering both variability with respect to angle of incidence (Figure 4a) and pump intensity (Figure 4b). Conversion efficiency here is defined as the ratio of third‐harmonic output power to the fundamental input power. We note how the simulation correctly predicts the sub‐quadratic scaling of THG efficiency with pump intensity only when Equations (1) and (2) are solved simultaneously with the finite element method. Omitting Equation (1) or solving the two equations in segregated steps produces the near‐quadratic trend expected from a simplified theory (Figure 4b, dashed, blue line).

**FIGURE 4:**
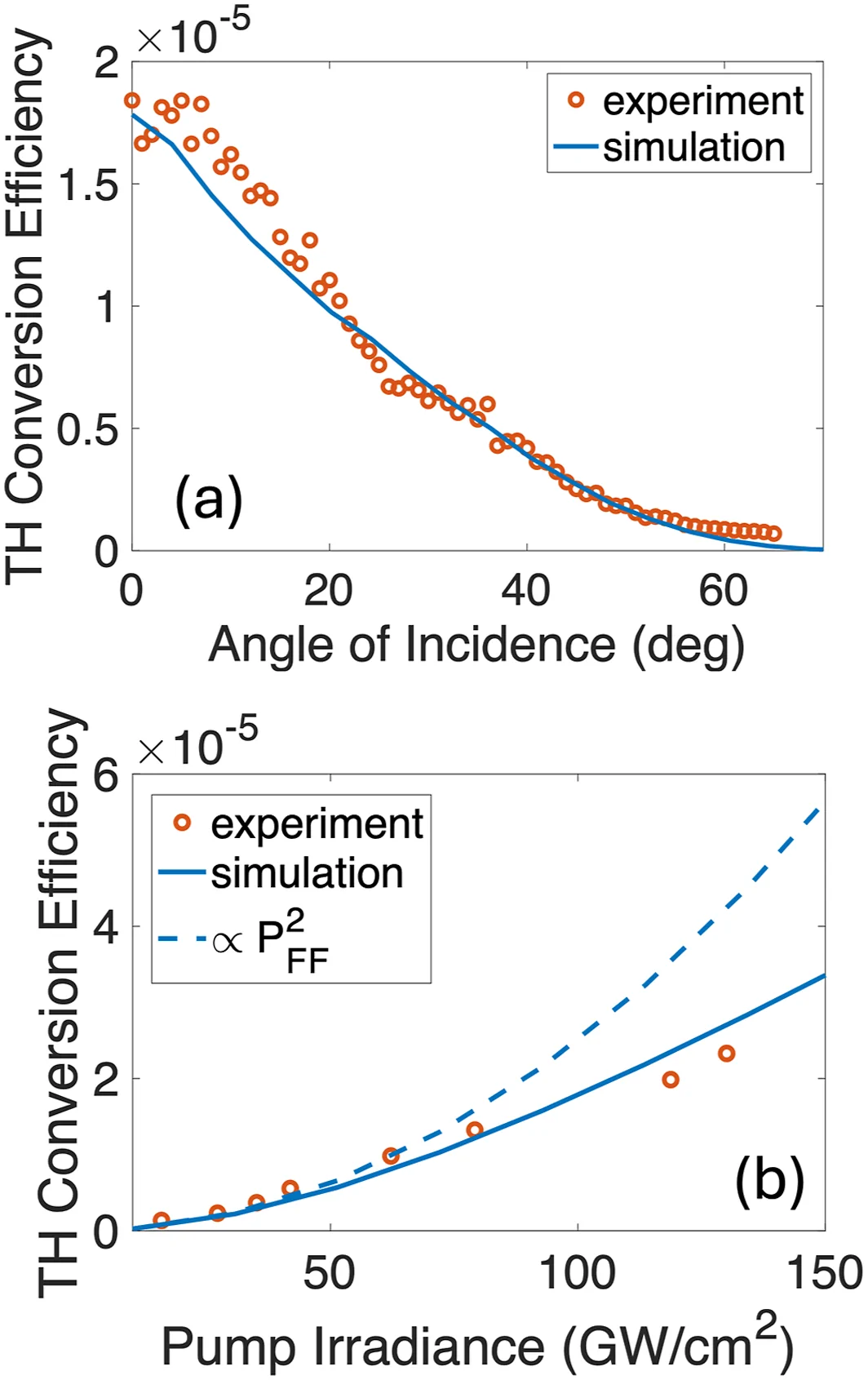
(a) Measured (red markers) and simulated (blue line) TH conversion efficiency of the 10 period αSi/ITO multilayer stack as a function of the angle of incidence for a pump wavelength at 1250 nm and 100 GW/cm^2^ TM‐polarized pump intensity. (b) Measured (red markers) and simulated (solid blue line) TH conversion efficiency for increasing pump intensity and fixed pump wavelength at 1250 nm for normal incidence. Dashed blue line shows the predicted power scaling law of THG without including the terms in Equation (1).

The model developed with the finite element method through Equations (1) and (2) assumes a continuous wave pump tuned at 1250 nm and is not sufficient to reproduce the TH response in a pulsed regime, nor to include nonlocal effects that might be appreciated in a time domain dynamics. For these reasons, we also use a time‐domain theory that fully couples matter dynamics to the macroscopic Maxwell's equations. Examples of the method can be found in refs. [32, 43]. Concretely, we use a hydrodynamic‐Maxwell model designed to capture both linear and nonlinear optics of conducting oxides and amorphous silicon. The model simultaneously accounts for surface, magnetic, and bulk nonlinear sources from free and bound charges; retains linear and nonlinear dispersions; includes nonlocal corrections from pressure and viscosity; allows the free‐electron effective mass in ITO to change under strong absorption; and can incorporate pump depletion when local fields become sufficiently large.

The standard macroscopic treatment of conducting oxides retains only the Drude free‐electron term to describe the dielectric function. We instead model ITO's dielectric function as the sum of a Drude background and a single ultraviolet Lorentzian resonance, so the local permittivity acquires an added bound‐electron contribution:

(3) $$\varepsilon_{ITO}\left(\omega \right)=1-\frac{\omega_{p}^{2}}{\omega^{2}+i\gamma_{f}\omega }-\frac{\omega_{p,b}^{2}}{\omega^{2}-\omega_{0,b}^{2}+i\gamma_{b}\omega }$$

In Equation (3), $\omega_{p}^{2}=\frac{n_{0,f}e^{2}}{m_{f}^{*}}$ is the plasma frequency; $n_{0,f}$ the free electron density; *e* the electronic charge; $\gamma_{f}$ the free electron damping coefficient; and $m_{f}^{*}$ the temperature‐dependent effective free electron mass; $\omega_{p,b}$ is the bound electron plasma frequency, defined similarly to the free electron counterpart; $\omega_{0,b}$ is the resonance frequency; and $\gamma_{b}$ the bound electron damping coefficient. Including the Lorentzian term injects a crucial, dynamic degree of freedom into the nonlinear response: it produces an intrinsic dispersive resonant nonlinearity in the ITO that is absent from purely Drude descriptions and that can materially affect harmonic generation. Practically, two nonlinear channels can compete. One is the resonant dispersion‐mediated response tied to the Lorentzian bound resonance; the other is the nonlinearity that arises when free carriers heat and the effective mass (and hence the Drude response) evolves with pump intensity. Their relative importance depends on spectral detuning, material dispersion, and incident pump intensity.

Therefore, this combined Drude–Lorentz description modifies both linear phase and frequency dependence of the nonlinear susceptibility, with direct consequences for harmonic‐generation performance: it alters phase matching, bandwidth, and conversion efficiency and can either enhance or suppress harmonics produced by free‐carrier mechanisms. In the usual approach, only the Drude portion of the dynamics is considered, whereas the Lorentzian response is typically ignored by using an expression of the type $\varepsilon_{ITO}\left(\omega \right)=\varepsilon_{\infty }-\frac{\omega_{p}^{2}}{\omega^{2}+i\gamma_{f}\omega }$, where $\varepsilon_{\infty }$ is a constant. This risks misattributing spectral features or the scaling of harmonic yield with pump power. Including it provides a more faithful, predictive dielectric function across the experimental spectral range and helps identify which physical pathway (resonant bound‐electron nonlinearity or free‐carrier heating) dominates in each design, enabling more informed choices to maximize nonlinear efficiency while controlling dispersion and loss. Just as importantly, a full description of dispersion that follows Equation (3) fully preserves the dynamics of momentum transfer between fields and charges. Unlike ITO, silicon is modeled as a dielectric composed of three Lorentzian resonances, each carrying its own intrinsic dispersive nonlinear response. Building on these assumptions, we formulate coupled time‐domain equations of motion that yield two distinct polarization channels: one from free carriers and one from bound electrons, written separately for each material. We neither impose a preformed dielectric constant, nor introduce pseudo‐singular denominators due to the ENZ crossing point to rationalize large conversion efficiencies. Instead, fitting a retrieved local dielectric function only fixes physical parameters (damping rates, effective masses, and carrier densities) that will populate the dynamical equations; the linear dielectric constant can be reconstructed *a posteriori* if desired.

The polarization equations include the lowest‐order bulk, surface, and magnetic nonlinear sources, which can account simultaneously for even and odd harmonic generation in centrosymmetric materials such as ITO and αSi. These terms are adapted from our prior work on metals and semiconductors and used here to capture temperature‐ and time‐dependent carrier dynamics. Plasma and resonance frequencies in both ITO and Si are allowed to evolve with temperature and intensity. We assume the total number of free carriers remains constant, although their local density can vary in space and time, so absorption‐driven changes in effective mass and density feedback self‐consistently into linear dispersion and nonlinear coupling. This unified formulation exposes competing nonlinear channels, that is, resonant Lorentzian contributions from bound electrons in Si and ITO, free‐carrier dynamics in ITO, nonlocal and surface effects, and lets their relative importance naturally emerge from the dynamics.

Equations (4) and (5) govern the time‐domain dynamics of free and bound charges, respectively:

(4) $${\ddot{P}}_{f}+{\tilde{\gamma }}_{f}{\dot{P}}_{f}=\frac{n_{0,f}e^{2}\lambda_{0}^{2}}{m_{f}^{*}\left(T_{e}\right)c^{2}}E-\frac{e\lambda_{0}}{m_{f}^{*}\left(T_{e}\right)c^{2}}E\left(\nabla \cdot P_{f}\right)+\frac{e\lambda_{0}}{m_{f}^{*}\left(T_{e}\right)c^{2}}{\dot{P}}_{f}\times H+\frac{3E_{F}}{5m_{f}^{*}\left(T_{e}\right)c^{2}}\left(\nabla \left(\nabla \cdot P_{f}\right)+\nabla^{2}P_{f}\right)-\frac{1}{n_{0,f}ec}\left[\left(\nabla \cdot {\dot{P}}_{f}\right){\dot{P}}_{f}+\left({\dot{P}}_{f}\cdot \nabla \right){\dot{P}}_{f}\right],$$

(5) $${\ddot{P}}_{bj}+{\tilde{\gamma }}_{bj}{\dot{P}}_{bj}+{\tilde{\omega }}_{0,bj}^{2}P_{bj}+P_{bj,NL}=\frac{n_{0,bj}e^{2}\lambda_{0}^{2}}{m_{bj}^{*}c^{2}}E+\frac{e^{2}\lambda_{0}}{m_{bj}^{*}c^{2}}\left(P_{bj}\cdot \nabla \right)E+\frac{e^{2}\lambda_{0}}{m_{bj}^{*}c^{2}}{\dot{P}}_{bj}\times H.$$

In the free‐carrier equation (Equation 4), *T*_*e*_ denotes the electron temperature; in the bound‐carrier equation (Equation 5) $m_{b}^{*}$ and $n_{0,b}$ denote the bound‐electron mass and density, both assumed to be constant; $P_{bj}$ represents the *j*th bound‐electron polarization for a given material, and $P_{bj,NL}=\alpha P_{bj}P_{bj}-\beta \left(P_{bj}\cdot P_{bj}\right)P_{bj}+....$ is the nonlinear polarization. The tensors *α* and β encode crystal symmetry (for centrosymmetric media such as ITO, αSi, and noble metals *α* = 0). The third‐order nonlinear coefficient is written for an isotropic medium but can be generalized to other symmetries.

This coupled dynamical formulation intentionally omits any presupposed dielectric constant or refractive index: *causality demands that linear and nonlinear dispersions, surface harmonic sources, nonlocal pressure and viscous terms, magnetic contributions, and convective terms all emerge from the charge dynamics*. In particular, the free‐electron polarization in ITO mirrors that of noble metals except that the relevant free‐carrier parameters (e.g., the plasma frequency) depend on *T*_*e*_. The same framework can readily accommodate time‐varying free‐carrier densities produced by interband excitation in metals or semiconductors. In addition, the theory infrastructure makes it clear that relying exclusively on the two‐temperature model nonlinearity (nonlinear free electron mass) is insufficient to treat the vast wavelength range that must be considered to properly model the electrodynamics of the problem.

Term by term, Equation (4) groups Coulombic contributions, the magnetic Lorentz force, nonlocal pressure and viscosity terms, and convective nonlinearities. Equation (5) is dominated by the bound‐electron resonance (bound plasma frequency) and includes surface and magnetic Lorentz contributions. Because both equations retain lowest‐order bulk, surface, and magnetic nonlinear sources, the model lets competing nonlinear channels (resonant bound responses, free‐carrier heating and mass changes, nonlocal spatial dispersion, and surface harmonics at oblique incidence) reveal themselves self‐consistently from the dynamics rather than from imposed singularities or fitted denominators.

We model hot carriers with a dynamical effective‐mass picture that captures the essential physics of a full two‐temperature model while remaining compact and transparent. For the modest electron temperatures relevant here (a few thousand °K), we approximate the ITO effective mass with the temperature set by absorption and the current density emerging from the free‐carrier equation of motion:

(6) $$m_{f}^{*}\left(T_{e}\right)\approx m_{0}^{*}+\alpha K_{B}T_{e}=m_{0}^{*}+\alpha K_{B}\Lambda \int \int J\cdot Edr^{3}dt$$

For convenience, this relation is simplified by defining $J=\sigma_{0}E$, where $\sigma_{0}$ is a constant to be determined, which allows one to write in simplified form:

(7) $$\frac{n_{0,f}e^{2}\lambda_{0}^{2}}{m_{f}^{*}\left(T_{e}\right)c^{2}}E\approx \frac{n_{0,f}e^{2}\lambda_{0}^{2}}{m_{0}^{*}c^{2}}E-\tilde{\Lambda }\left(E\cdot E\right)E+{\tilde{\Lambda }}^{2}{\left(E\cdot E\right)}^{2}E+...$$

Using Equations (6) and (7), the free electron plasma frequency in Equation (4) is simplified to an intensity dependent law with a single fitted constant that incorporates dispersion, absorption strength, interaction volume, and pulse duration. Using this temperature‐dependent effective mass, the dominant Coulomb term in the free‐carrier equation acquires an explicit intensity‐dependent shift that appears as a redshift of the plasma frequency and generates cubic, quintic, and higher order nonlinearities that can become important with increasing peak power densities. Geometric factors and the pulse temporal overlap are folded into scalar coefficients, so that our simulations account for an intensity‐driven free‐carrier dynamic. The bound‐electron equation of motion remains unchanged. We then solve the free‐ and bound‐electron equations together with the temperature‐dependent effective‐mass and sum the resulting polarizations $P_{Total}=P_{f}+P_{b}$ which is then inserted into Maxwell's equations. Details of the integration scheme and parameter values are summarized in Supporting Information S1.

This approach retains the dominant physical route by which absorption alters dispersion and nonlinearity in the ITO schematically as follows: (i) absorption heats carriers; (ii) effective mass changes; (iii) the plasma frequency redshifts; and (iv) intensity dependent behavior follows. Therefore, we preserve the mechanisms required to capture sub‐quadratic harmonic scaling, phase locking [40], and pump‐depletion effects when fields become large, while avoiding the complexity of a full two‐temperature problem solver. Higher order corrections and a full two‐temperature treatment can be reintroduced when experimental conditions demand it.

Figure 5 compares the experimentally measured transmitted third‐harmonic spectra (Figure 5a) with the spectra predicted by our model (Figure 5b) using a TM‐polarized incident pump field. The solid black trace in Figure 5b shows the transmitted THG conversion efficiency from the simulation; its amplitude matches the measured values to within experimental uncertainty. This close qualitative and quantitative agreement indicates that the time‐domain, hydrodynamic treatment, with coupled free‐ and bound‐electron dynamics, temperature‐dependent effective mass, and retained surface, magnetic, and nonlocal contributions, faithfully captures the dominant physical phenomenology that determine harmonic spectral response and harmonic efficiencies. Reproducing both spectral shape and efficiency confirms the model's ability to predict intensity‐dependent scaling and phase‐locking effects, and supports its use for designing LENZ multilayers that optimize conversion while preserving pump transparency.

**FIGURE 5:**
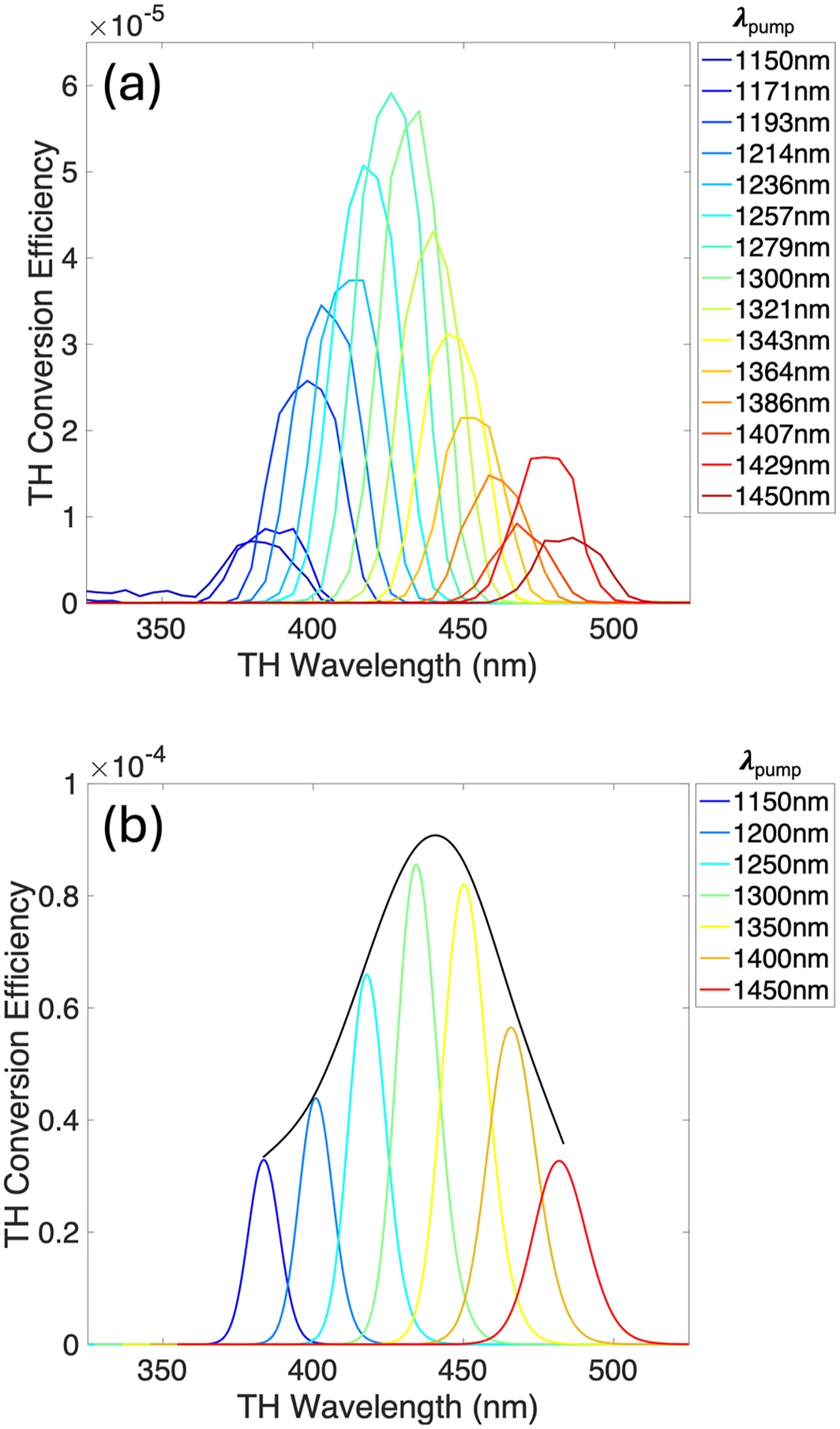
(a) Measured and (b) simulated TH conversion efficiency of the 10 period Si/ITO multilayer stack as a function of the pump wavelength for 300 GW/cm^2^ pump irradiance.

Finally, Figure 6 shows the simulated third‐harmonic conversion efficiency as a function of wavelength for two peak incident power densities. At low intensity, a THG peak of order 10^−8^ (right axis) appears near ITO's ENZ wavelength, which remains essentially fixed around 1260 nm. By contrast, raising the peak power density to 300 GW/cm^2^ drives a ∼60 nm redshift of the ITO plasma edge and boosts the predicted conversion efficiency in excess of three orders of magnitude (left axis). The plot illustrates two coupled effects. At weak excitation the ENZ resonance delivers modest field enhancement with negligible spectral shift, so conversion stays small. At higher intensities, absorption heats carriers, the effective mass and plasma frequency move, and the resonant condition migrates spectrally; this dynamical redshift is accompanied by strong nonlinear gain seen in the high‐power curve. The result highlights how intensity‐dependent dispersion in LENZ media can convert modest ENZ enhancement into orders‐of‐magnitude gains in harmonic yield, while also highlighting the trade‐offs introduced by pump‐driven material shifts and transparency.

**FIGURE 6:**
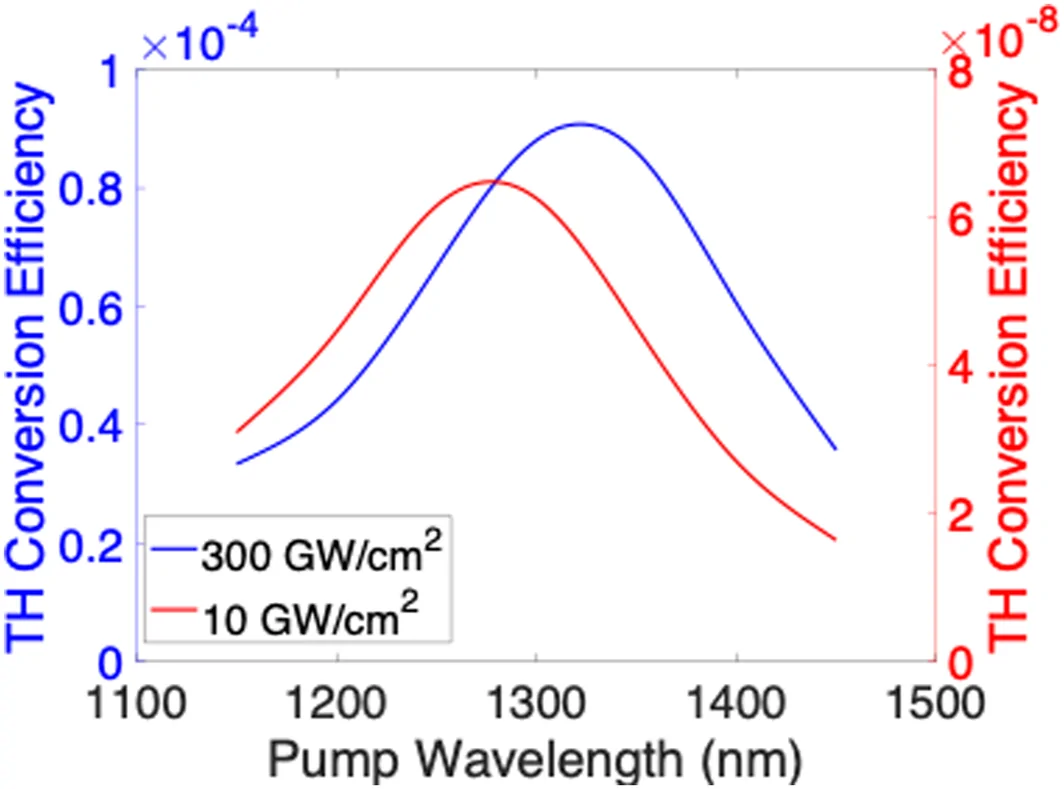
Simulated TH conversion efficiency response as a function of wavelength for pump irradiances of 10 GW/cm^2^ (red line—right axis) and 300 GW/cm^2^ (blue line—left axis).

## 4 Foundational Considerations on the Effective Mass Coefficient and Role of αSi

A recurring question in modeling hot‐electron nonlinearities in TCOs concerns the wavelength dependence of the phenomenological coefficient $\tilde{\Lambda }$ appearing in the intensity‐dependent effective mass (Equation 7). Although our full hydrodynamic formulation does not rely on this simplified ansatz, it is useful to clarify the physical meaning of $\tilde{\Lambda }$, its expected dispersion, and how it behaves when the ENZ crossing point itself becomes intensity‐dependent. This subsection summarizes the conceptual framework that motivates our modeling choices and explains how the nonlinear dynamics should be interpreted.

### 4.1 Physical Origin of $\tilde{\Lambda }$ and Its Dispersion

In a Drude–Lorentz TCO, such as ITO or AZO, the dominant hot‐electron nonlinearity arises from absorption‐driven changes in the free‐carrier effective mass as in Equation (6). Because the plasma frequency satisfies $\omega_{p}^{2}\;\propto \;\frac{1}{m^{*}\left(T_{e}\right)}$, any temperature‐induced mass increase produces a redshift of the ENZ crossing point, and a corresponding modification of the dielectric function. The coefficient $\tilde{\Lambda }\left(\lambda \right)$ is therefore proportional to the spectral derivative $\tilde{\Lambda }\;\propto \;\frac{\partial \varepsilon \left(\lambda \right)}{\partial T_{e}}$, which is largest where the dielectric function is most sensitive to changes in $m^{*}\left(T_{e}\right)$. This occurs in the spectral region where the Re(ε)=0 and where the Drude and Lorentzian contributions interfere most strongly. Consequently, $\tilde{\Lambda }\left(\lambda \right)$ is not spectrally flat. Instead, in the low‐intensity (linear) regime it exhibits a Lorentzian‐like peak centered at the static ENZ wavelength $\lambda_{ENZ,0}$. The width of this peak is set by the spectral region over which ∂ε/∂ω is large and can range from a few tens to a few hundred nanometers. A reasonable phenomenological form one may adopt is $\tilde{\Lambda }\left(\lambda \right)∼{\tilde{\Lambda }}_{0}{\left[1+{\left(\frac{\lambda -\lambda_{ENZ,0}}{\Gamma }\right)}^{2}\right]}^{-1}$ for ITO in the near‐infrared. For example, the function is plotted below for *Γ* = 100 and $\lambda_{ENZ,0}=1230\;\text{nm}$. However, in the calculation the shape and peak location of $\tilde{\Lambda }\left(\lambda \right)$ are dictated dynamically by a combination of material dispersion and peak power density.

### 4.2 Behavior of $\tilde{\Lambda }$ Outside the ENZ Condition

The spectral shape of $\tilde{\Lambda }\left(\lambda \right)$ has direct consequences for nonlinear dynamics. If the pump wavelength $\lambda_{\text{pump}}$ is initially tuned below the ENZ wavelength, at low intensity the pump lies on the *blue side* of the Lorentzian peak in $\tilde{\Lambda }\left(\lambda \right)$ shown in Figure 7. As intensity increases, carrier heating increases, redshifting the ENZ wavelength to longer values. The pump therefore effectively moves *away* from the peak of $\tilde{\Lambda }\left(\lambda \right)$. The effective nonlinearity *decreases monotonically* with intensity. The nonlinear enhancement is therefore most pronounced at low intensity and gradually weakens as the ENZ condition moves away from the excitation wavelength, with no maximum occurring at intermediate intensities.

**FIGURE 7:**
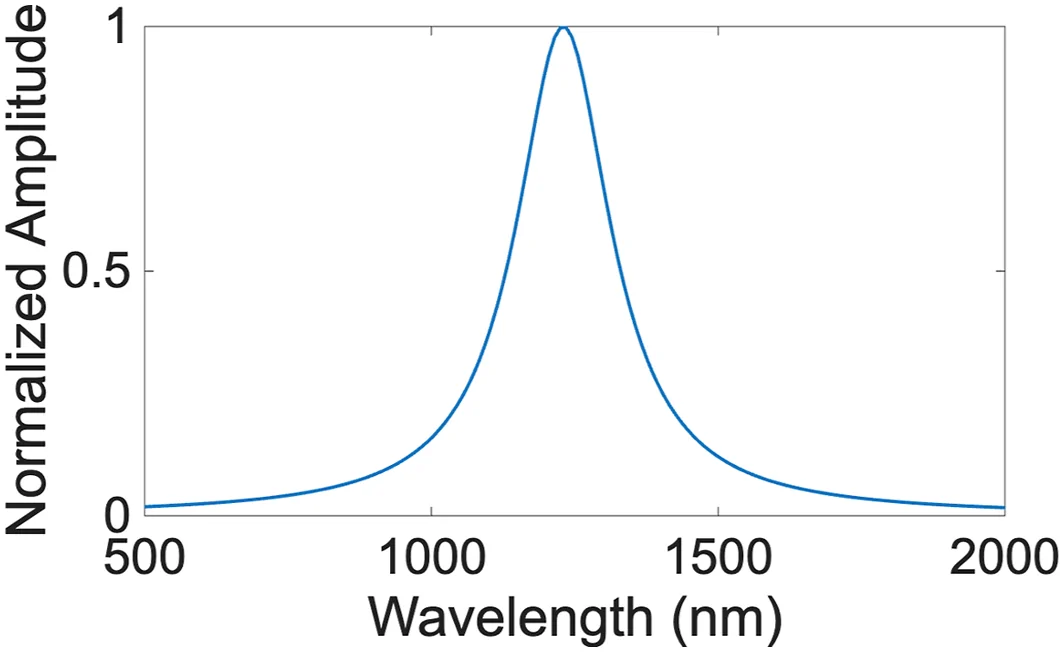
Illustration of the normalized phenomenological dispersion of the hot‐electron nonlinear coefficient $\tilde{\Lambda }\left(\lambda \right)$.

If the pump is initially tuned *above* the ENZ wavelength, the situation reverses. At low intensity, the pump lies on the *red side* of the Lorentzian. As intensity increases and ENZ redshifts, the pump moves *toward* the peak of $\tilde{\Lambda }\left(\lambda \right)$. When $\lambda_{ENZ}\left(I\right)\;\approx \;\lambda_{\text{pump}}$, the effective nonlinearity reaches a *maximum*. At still higher intensities, the ENZ moves past the pump, and $\tilde{\Lambda }\left(I\right)$ decreases again. Thus $\tilde{\Lambda }\left(I\right)$ is *bell‐shaped*. In other words, it is small at low intensity, rising sharply as ENZ approaches the pump, peaking when the pump and ENZ wavelengths coincide, decreasing once the ENZ overshoots. This behavior naturally produces THG‐scaling that deviates from expected quadratic behavior at intermediate intensities, saturation at high intensities, and an intensity‐dependent optimal pump wavelength. This is precisely the behavior observed in Figure 6.

### 4.3 How to Choose $\tilde{\Lambda }$ in a Dynamical Simulation

A key point is that $\tilde{\Lambda }$ *should be chosen from the linear regime*, based solely on the pump detuning from the *static* ENZ wavelength. Once this initial value is fixed, the nonlinear dynamics must be allowed to evolve self‐consistently through the temperature‐dependent effective mass and the hydrodynamic equations. One should not manually update $\tilde{\Lambda }\left(\lambda \right)$ as the ENZ shifts; doing so would double‐count the same physical mechanism and lead to significant inaccuracies. Thus, the correct procedure is as follows: (i) Determine $\tilde{\Lambda }\left(\lambda_{p}\right)$ from the low‐intensity dispersion, transmission or reflection curve, using the Lorentzian‐like form centered at $\lambda_{ENZ,0}$; (ii) hold $\tilde{\Lambda }\left(\lambda_{p}\right)$ fixed throughout the simulation; (ii) allow the dynamical evolution of $m^{*}\left(T_{e}\right)$ to redshift the ENZ wavelength and modify the dielectric function; (iv) let the hydrodynamic‐Maxwell coupling determine the resulting THG efficiency, spectral shifts, and scaling behavior. This approach ensures that the nonlinear response arises from the correct physical pathway, namely absorption‐driven carrier heating and the resulting modification of the Drude term, rather than from an artificially moving, unscaled, and possibly physically unrelated nonlinear coefficient.

### 4.4 Implications for TLENZ Multilayers and Comparison With Thick ITO Films

The behavior of $\tilde{\Lambda }$ also clarifies why TLENZ multilayers may outperform an isolated ITO film. In a thick ITO film, the ENZ wavelength may lie deep in the opacity region. The pump may not be able to reach the region where $\tilde{\Lambda }$ is large. In contrast, in a TLENZ multilayer, the ENZ condition is preserved while maintaining high pump transparency, allowing the pump to access the spectral region where $\tilde{\Lambda }$ is maximized, while maintaining control of bandwidth and transmission maximum. Finally, under strong pumping, the dynamic ENZ redshift moves the resonance through the pump wavelength, enabling the bell‐shaped nonlinear enhancement described above. Thus, the interplay between the static dispersion of $\tilde{\Lambda }$ and the dynamic migration of the ENZ crossing point is central to understanding the orders‐of‐magnitude enhancement observed in Figure 6. As an example, in Figure 8 below, we show both transmitted THG efficiency for peak power densities of 300 and 600 GW/cm^2^, for an isolated, 270nm‐thick ITO layer. The curves are redshifted relative to each other because of increased effective mass due to temperature variations of the free electron gas.

**FIGURE 8:**
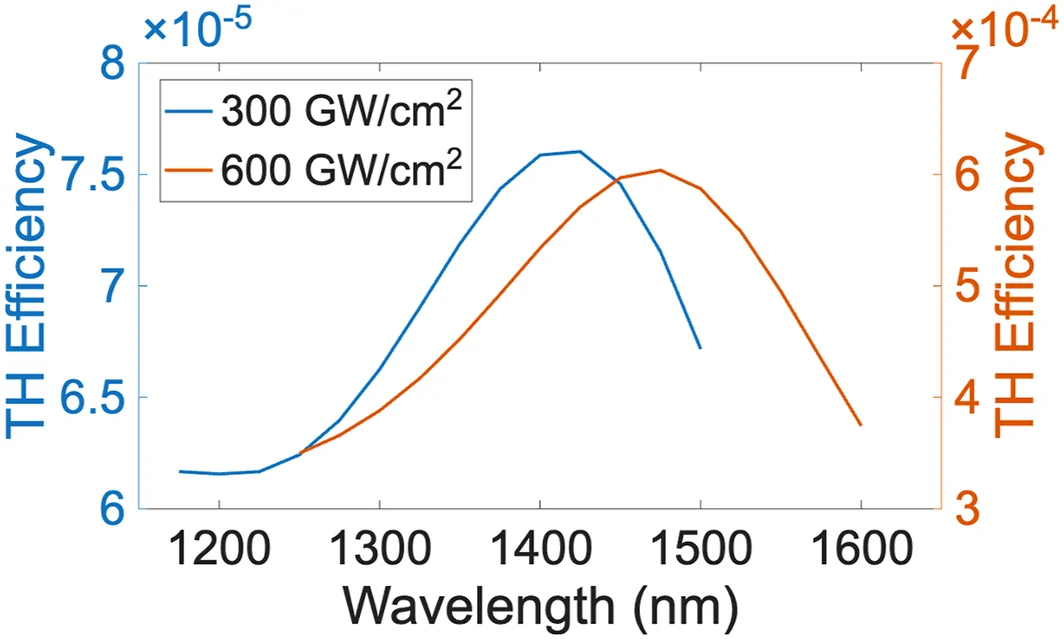
Transmitted TH efficiency for 300 (blue curve, left axis) and 600 GW/cm^2^ (red curve, right axis) from a suspended uniform ITO film 270 nm thick. The redshift occurs because the effective free electron mass increases, pushing the plasma frequency toward longer wavelengths. Here, we assume $\tilde{\Lambda }\left(\lambda \right)=10^{-9}$.

### 4.5 Role of the αSi Layer

The amorphous‐silicon (αSi) layers in the TLENZ stack play a more active role in the nonlinear response than their nominally dielectric character might suggest. Although the ENZ‐driven enhancement is primarily associated with the ITO layers, the αSi sublayers introduce additional resonant and dispersive nonlinear channels that become relevant once the pump wavelength, field distribution, and multilayer geometry are considered together.

A very important aspect of the Si layers lies in its influence on the angular response of the THG (Figure 4a), which should not be interpreted as arising from a purely LENZ‐dominated mechanism. For TM excitation, the longitudinal field component vanishes at normal incidence, where the response is instead mainly driven by transverse field components and by the sizable bound‐electron χ^(3)^ contribution of the αSi layers. At finite incidence angles, the longitudinal field becomes progressively more relevant, allowing the LENZ‐mediated channel to contribute to the nonlinear response. Thus, the TLENZ stack operates as a hybrid transverse–longitudinal nonlinear platform, where the αSi‐driven response dominates near normal incidence, whereas the LENZ contribution helps sustain a broad angular acceptance compared with a single homogeneous χ^(3)^ layer.

### 4.6 Bound‐Electron Resonance, χ^(3)^ Enhancement, and Comparison With ITO

Amorphous silicon exhibits a strong interband absorption resonance near ∼ 350 nm, corresponding to the first Lorentzian oscillator in its dielectric response. According to Miller's rule and standard harmonic‐oscillator scaling arguments [42, 44], the strengths of the third‐order susceptibilities χ^(3)^ (*ω*) and χ^(3)^ (3*ω*) are closely tied to the proximity of the driving frequency to such bound‐electron resonances.

In Figure 9 above, we display the calculated THG conversion efficiencies from the αSi/ITO multilayer we studied, an equivalent 10‐period stack that begins with ITO, and an isolated ITO layers. The figure illustrates the importance of dispersion and geometry. χ^(3)^ (*ω*) is relatively flat in the near‐infrared, but χ^(3)^ (3*ω*) grows substantially as the third harmonic approaches the ultraviolet resonance (see Supporting Information S1 on αSi third order susceptibilities). For pump wavelengths above ∼800 nm, the generated third harmonic lies between 260 and 400 nm, in proximity of the αSi's bound‐electron resonances, enhancing χ^(3)^ (3*ω*). Thus, even though the pump is far from any αSi resonance, the third harmonic is not. This creates a natural pathway for bound‐electron‐driven THG originating in the αSi layers. Extending the calculation down to 800 nm shows the pivotal role geometry can play in enhancing conversion efficiency beyond what is possible at the ENZ crossing point of the ITO layer, highlighting the multiple benefits one can extract from a hybrid approach. Several conclusions may then be drawn: (i) The multilayer can produce THG conversion efficiencies comparable or larger than the isolated ITO slab that contains similar total ITO thickness. This confirms that the multilayer geometry, not simply the amount of ITO, drives the enhancement; (ii) the broadband transmission enabled by the αSi layers allows the pump to reach the ENZ region without being absorbed, something not easily achieved in a thick ITO film; (iii) the field localization in αSi contributes additional nonlinear polarization at 3*ω*, especially when the third harmonic lies near the αSi resonance; and (iv) the combined effect is a hybrid nonlinear response: free‐electron ENZ‐enhanced nonlinearity in ITO, bound‐electron χ^(3)^ (3*ω*) enhancement in αSi, and cavity‐mediated field redistribution across the multilayer.

**FIGURE 9:**
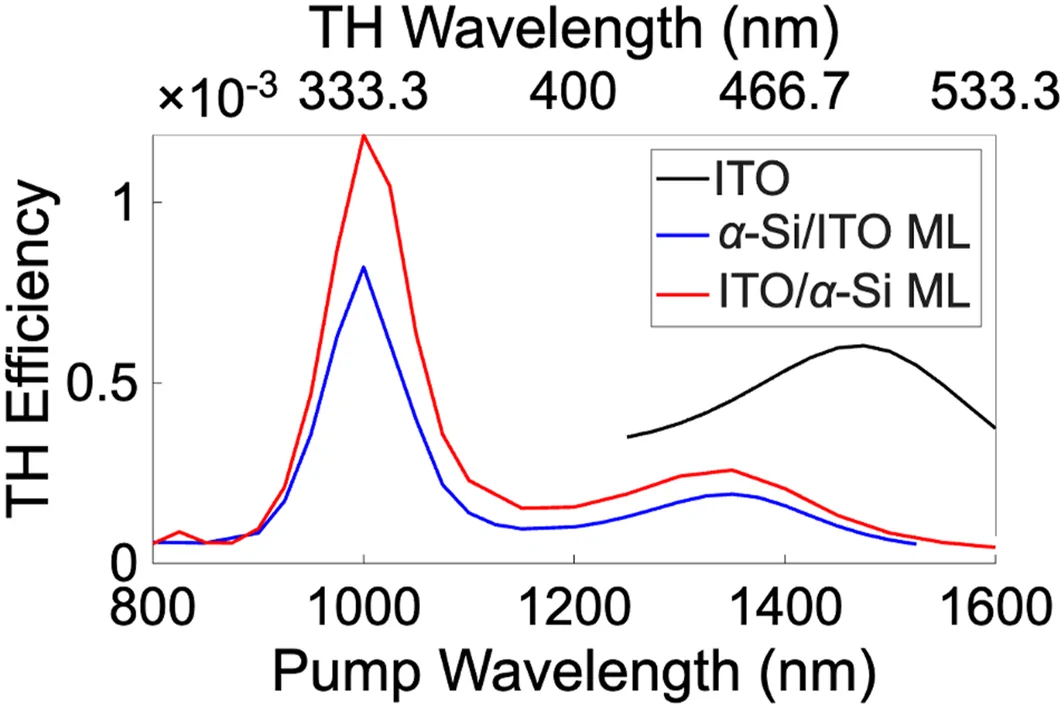
Calculated transmitted TH efficiency for two multilayers, as indicated in the figure, and an isolated ITO layer that contains the same total thickness of ITO. The plot clearly shows that a hybrid approach that considers geometrical considerations can improve conversion efficiency compared to ENZ effects alone. $\tilde{\Lambda }\left(\lambda \right)=10^{-9};\;{\tilde{\beta }}_{1}={\tilde{\beta }}_{2}={\tilde{\beta }}_{3}=3\times 10^{-8}$.

This hybridization is absent in the single ITO slab, which is both more absorptive and lacks the resonant χ^(3)^ (3*ω*) contribution from αSi. Therefore, the αSi layers in the TLENZ stack are not passive spacers. They shape the broadband transmission window, redistribute the pump field across the multilayer, provide a resonantly enhanced χ^(3)^ (3*ω*) channel for THG, and cooperate with the ITO ENZ response to produce a nonlinear yield that may exceed either material alone. The result is an interactive nonlinear platform in which the free‐electron dynamics of ITO and the bound‐electron resonances of αSi jointly determine the THG efficiency, spectral shape, and intensity scaling.

Nonlocal effects play different roles in the systems we are considering. They impart a considerable blueshift to the dispersion curve [19] of thin layers, which offset and limit the redshift triggered by changes to the effective mass. In contrast, nonlocal effects are negligible for thick ITO layers, leaving the effective mass triggered redshift to dominate. This is the reason why the multilayer stacks display THG peaks that have less pronounced redshifts compared to the isolated ITO layer (Figure 9 above).

### 4.7 Interplay With ITO: Free‐Versus Bound‐Electron Nonlinearities

In the ITO layers, the dominant nonlinear mechanism near ENZ is the free‐electron channel, driven by absorption‐induced changes in the effective mass and the resulting redshift of the plasma frequency. This mechanism is strongest when the pump is tuned near the ENZ wavelength, where the longitudinal field enhancement is maximal. In contrast, αSi contributes primarily through bound‐electron nonlinearities, which are dispersive and resonantly enhanced at 3ω. The relative importance of these two channels depends on: (i) the pump detuning from the ENZ condition, (ii) the degree of field localization in each sublayer, (iii) the spectral position of the third harmonic relative to the αSi resonance, and (iv) the thickness of the αSi layers, which controls the multilayer's broadband transmission window. When the pump is tuned close to ENZ, the ITO free‐electron nonlinearity dominates the overall THG yield. However, as the field becomes increasingly localized in the αSi layers, either due to cavity effects, interference, or the specific thickness ratio, the αSi contribution becomes non‐negligible, especially because its χ^(3)^ (3*ω*) is large in the UV. This, coupled with phase‐locking and survival of the inhomogeneous solution of the wave equation [41, 42, 45], leads to high conversion efficiencies. An example of the interplay between free and bound electron nonlinearities can be found in the Supporting Information S1.

## 5 Conclusions

We demonstrate transparent field‐enhanced third‐harmonic generation in a ten‐period αSi/ITO TLENZ multilayer. The fabricated stack preserves high pump transmission, with measured transmittance of the order of 50% over a broad spectral range, while supporting longitudinal field enhancement and sizable THG emission. Both finite‐element simulations and a fully coupled time‐domain hydrodynamic model reproduce the measured THG amplitude and spectral shape, confirming the relevance of the physical mechanisms included in the description.

The theoretical analysis shows that the nonlinear response cannot be assigned to a single channel. TLENZ anisotropy enhances the longitudinal field while keeping the pump within a relatively high‐transmission spectral window. At the same time, the αSi layers play an important role through their bound‐electron nonlinear response: although the pump is far from the αSi resonance, the generated third harmonic lies in the ultraviolet spectral region where the αSi χ^(3)^ contribution is enhanced. Therefore, the observed THG results from the hybridization of free‐electron ENZ‐enhanced nonlinearity in ITO and bound‐electron χ^(3)^ response in αSi, mediated by the multilayer field distribution. Within the investigated conditions, the TLENZ multilayer produces a THG conversion efficiency comparable to that of isotropic ENZ films while offering significantly higher pump transmission. This highlights the main advantage of the platform: not merely maximizing the nonlinear efficiency, but sustaining strong nonlinear generation under more transparent pump conditions. TLENZ multilayers therefore emerge as a designable platform for transparent nonlinear nanophotonics, combining field enhancement, reduced pump losses, and the possibility to tailor nonlinear dynamics through multilayer engineering. Their high pump transmission may also make them attractive for future cavity integration and for mitigating absorption‐induced heating, although dedicated studies will be required to quantify these potential advantages.

## Author Contributions

**Wallace Jaffray:** data curation, formal analysis, methodology, investigation, writing – original draft. **Sven Stengel:** investigation, methodology, formal analysis, data curation, writing – original draft. **Domenico de Ceglia:** conceptualization, investigation, validation, formal analysis, writing – original draft. **Neset Akozbek:** methodology, data curation. **Filippo Capolino:** conceptualization, writing – original draft, formal analysis, validation. **Marcello Ferrera:** conceptualization, investigation, supervision, data curation, writing – original draft, funding acquisition. **Michael Scalora:** conceptualization, methodology, formal analysis, data curation, writing – original draft. **Maria Antonietta Vincenti:** conceptualization, investigation, funding acquisition, writing – original draft, methodology, formal analysis, supervision.

## Funding

M.F. acknowledges financial support from EPSRC project ID EP/X035158/1; AFOSR (EOARD) under award no. FA8655‐23‐1‐7254; and EPSRC/STFC/NSERC under UK‐Canada Quantum for Science Research Collaborations OPP640‐APP47830. M.A.V. and D.d.C. thank the NATO Science for Peace and Security program (Grant #5984) for financial support. M.A.V. acknowledges financial support of Ministero dell'Istruzione, dell'Università e della Ricerca (PRIN Grant No. 2022YJ5AZH).

## Conflicts of Interest

The authors declare no conflicts of interest.

## Supporting information


Supporting Information S1

## Data Availability

The datasets generated and/or analyzed during the current study are available from the corresponding author upon reasonable request.
