# Supplementary material for: Third Harmonic Generation in Transparent Longitudinal Epsilon‐Near‐Zero Multilayers
Source: Nanophotonics. 2026 Jul 22;15(14):e70216. doi: 10.1002/nap2.70216 (PMC13642089; doi:10.1002/nap2.70216)
Supplement: Supplementary file 1 [file NAP2-15-e70216-s001.docx]

Supplementary Information

Wallace Jaffray, Sven Stengel, Domenico de Ceglia, Neset Akozbek, Filippo Capolino, Marcello Ferrera^[1]^, Michael Scalora and Maria Antonietta Vincenti

Third Harmonic Generation in Transparent Longitudinal Epsilon-Near-Zero Multilayers

**SUPPLEMENTARY NOTE 1: DIELECTRIC PERMITTIVITIES RETRIEVAL**

A single layer of ITO and amorphous silicon ($\alpha Si$) deposited following the procedure in Note 2 were analysed using a variable angle spectroscopic (300nm-1700nm) ellipsometer (Woollam, VASE) at different incident angles. Using a Lorentz-Drude dispersion model, the complex dielectric constants were retrieved and are shown in Fig.S1 (ITO) and Fig.S2 ($\alpha Si$).

**Fig. S1:** Dielectric permittivity of ITO retrieved from ellipsometric measurements.

**Fig. S2:** Dielectric permittivity of $\alpha Si$ retrieved from ellipsometric measurements.

**SUPPLEMENTARY NOTE 2: LENZ FABRICATION**

The ITO and amorphous Silicon layers combined into a ten periods multilayer stack are RF magnetron sputtered from an ITO target (In_2_O_3_/SnO_2_ 90/10wt %, purity 99.99%) and a Silicon target on transparent fused silica glass. The optical properties of deposited films depend on a number of processing conditions, including substrate temperature, RF sputtering power, deposition pressure, oxygen partial pressure, and post-annealing. The samples were fabricated at room temperature with a RF power of 100 W and Ar sputter pressure of 5 mTorr. The as-is sputtered ITO typically exhibits a plasma wavelength above 1800 nm. The samples were post-annealed at 400 C° in vacuum so that the plasma frequency would move toward 1300nm.

**SUPPLEMENTARY NOTE 3: EXPERIMENTAL SETUP**

**Fig. S3:** Experimental set up for nonlinear characterisation.

The third-harmonic generation experiments are driven by a Ti:sapphire laser, which pumps an optical parametric amplifier (OPA) to produce tuneable near-infrared pulses in the 1150–1450 nm range. The output beam is guided first through a half-wave plate and a polarizer, enabling control over the incident linear polarization, and then into a beam sampler for direct and continuous monitoring of input power. Subsequently, the main beam passes through a variable attenuator before going through a focusing lens to illuminate the sample with the desire local intensity. After interaction with the sample, the generated third-harmonic signal is collected and directed toward a detection stage consisting of appropriate spectral filtering followed by a calibrated photodiode for power measurements. The optical path is fully free-space, with wavelength-selective and polarization-control elements positioned as shown to ensure stable coupling conditions across the entire tuning range.

**SUPPLEMENTARY NOTE 4: ITO TRANSMITTANCE SPECTRA**

**Fig. S4:** Transmittance as a function of wavelength for an ITO film of different thicknesses. We assume normal incidence and glass substrate.

**SUPPLEMENTARY NOTE 5: HYDRODINAMIC-MAXWELL MODEL PARAMETERS**

In the Hydrodinamic-Maxwell approach described in the main text spatial and temporal derivative are performed with respect to the following scaled coordinates: $\tau=ct/\lambda_{0}; \tilde{x},\tilde{y},\tilde{z}=x/\lambda_{0},y/\lambda_{0},z/\lambda_{0}$, where $\lambda_{0}=1 \text{μ}\text{m}$ is a reference wavelength, *c* is the speed of light in vacuum. If we consider only the linear terms in Eq. (4) ans (5), a Fourier transform yields: $\varepsilon(\tilde{\omega})=1-\frac{\tilde{\omega}_{pf}^{2}}{\left( \tilde{\omega}^{2}+i\tilde{\gamma}_{f}\tilde{\omega} \right)}-\frac{\tilde{\omega}_{p2}^{2}}{\left( \tilde{\omega}^{2}-\tilde{\omega}_{02}^{2}+i\tilde{\gamma}_{b2}\tilde{\omega} \right)}+...$ where the dots indicate additional Lorentzian resonances. As mentioned above, ITO is fitted with one Drude function and one such Lorentzian, while the αSi is fitted with three bound electron resonances. The values used for ITO αSi are summarized in Tables S1 and S2, respectively.

Table S1. ITO fitting parameters

|  | $\tilde{\omega}_{p}$ | $\tilde{\omega}_{0}$ | $\tilde{\gamma}$ |
| --- | --- | --- | --- |
| Drude | 1.5785 | 0 | 0.15 |
| Lorentz | 8.8 | 5.5 | 0.01 |

Table S2. αSi fitting parameters

|  | $\tilde{\omega}_{p}$ | $\tilde{\omega}_{0}$ | $\tilde{\gamma}$ |
| --- | --- | --- | --- |
| Lorentz 1 | 5.97 | 2.9577 | 0.7931 |
| Lorentz 2 | 4.155 | 2.3891 | 0.6627 |
| Lorentz 3 | 5.973 | 2.9577 | 0.793 |

The local dielectric constant is recovered using the scaled frequency $\tilde{\omega}=1/\lambda$, where the wavelength is in microns, while $\tilde{\gamma}=\gamma_{0}\frac{\lambda_{0}}{c}$, $\tilde{\omega}_{0}^{2}=\omega_{0}^{2}\frac{\lambda_{0}^{2}}{c^{2}}$, $\tilde{\omega}_{p}^{2}=\frac{ne^{2}}{m_{0}^{2}}\frac{\lambda_{0}^{2}}{c^{2}}=\tilde{\omega}_{p0}^{2}\frac{\lambda_{0}^{2}}{c^{2}}$. The parameter $\tilde{\beta}_{j}\approx\omega_{0,bj}^{2}\lambda_{0}^{2}/\left( L^{2}n_{0bj}^{2}e^{2}c^{2} \right)$ is extracted from the nonlinear oscillator model. For αSi only geometry, THG conversion efficiency is proportional to $\tilde{\beta}_{j}^{2}\sim(L^{4}n_{0bj}^{4})^{-1}$. For our purposes it suffices to assume that $\tilde{\beta}_{j}$ is constant, and that $\tilde{\beta}_{1}\approx\tilde{\beta}_{2}\approx\tilde{\beta}_{3}$ for the three oscillators, given the proximity of the resonances, and similarly for the respective effective masses. *L* represents the classical length of a spring, which in our case takes on the value of either the lattice constant, or for a more microscopic approach, the diameter of the participating orbital extracted from quantum mechanical calculations of the wave functions.

**SUPPLEMENTARY NOTE 6: AMORPHOUS SILICON THIRD ORDER SUSCEPTIBILITIES**

**Fig. S5: (a)** Linear, complex dielectric response of αSi fitted using three Lorentzian resonances as detailed in Supplementary Note 5. This fit allows to go beyond the ellipsometry measurements of Fig. S2. Third order nonlinear dispersion for the pump **(b)** and the third harmonic signal **(c)**. These functions may be obtained directly from a nonlinear oscillator model.

**SUPPLEMENTARY NOTE 7: EFFECT OF FREE ELECTRON NONLINEARITY ON THG**

A doubling of the free electron nonlinearity in the multilayer geometry causes minimal changes to both peak location and curve bandwidths.

**Fig. S6:** Reflected and transmitted THG efficiencies from (αSi/ITO) x10 multilayer stack, for two different nonlinear free electron nonlinearities, as indicated in the figure. Nonlocal effects in the thin ITO layer temper the redshift observed in an ITO-only layer by blue-shifting the band structure.
